# Discovery pipeline for epigenetically deregulated miRNAs in cancer: integration of primary miRNA transcription

**DOI:** 10.1186/1471-2164-12-54

**Published:** 2011-01-21

**Authors:** Toby Hulf, Tennille Sibbritt, Erik D Wiklund, Saul Bert, Dario Strbenac, Aaron L Statham, Mark D Robinson, Susan J Clark

**Affiliations:** 1 Epigenetics Laboratory, Cancer Research Program, Garvan Institute of Medical Research, 384 Victoria Street, Darlinghurst, NSW 2010, Australia; 2 Department of Molecular Biology, Aarhus University, 8000 Aarhus C, Denmark; 3 Bioinformatics Division, Walter and Eliza Hall of Medical Research, 1G Royal Parade, Parkville, VIC 3052, Australia; 4 St Vincent's Clinical School, University of NSW, St Vincent's Hospital, Darlinghurst, Sydney, 2010, New South Wales, Australia

## Abstract

**Background:**

Cancer is commonly associated with widespread disruption of DNA methylation, chromatin modification and miRNA expression. In this study, we established a robust discovery pipeline to identify epigenetically deregulated miRNAs in cancer.

**Results:**

Using an integrative approach that combines primary transcription, genome-wide DNA methylation and H3K9Ac marks with microRNA (miRNA) expression, we identified miRNA genes that were epigenetically modified in cancer. We find miR-205, miR-21, and miR-196b to be epigenetically repressed, and miR-615 epigenetically activated in prostate cancer cells.

**Conclusions:**

We show that detecting changes in primary miRNA transcription levels is a valuable method for detection of local epigenetic modifications that are associated with changes in mature miRNA expression.

## Background

MiRNA genes are typically transcribed by RNA polymerase II into primary miRNA (pri-miRNA) transcripts, and transcription appears to be regulated in a similar process as traditional coding genes [1]. Pri-miRNA transcripts are long non-coding RNAs (ncRNA) with stem-loop secondary structures that contain precursor miRNAs (pre-miRNA). Pri-miRNA transcripts are cleaved co-transcriptionally by the enzyme Drosha into stem loop pre-miRNAs, which are exported to the cytoplasm by Exportin 5, and further processed by Dicer into mature miRNA that can be loaded in the RNA induced silencing complex (RISC). Pre-miRNA levels are low relative to pri- and mature miRNAs, suggesting that dicing is an efficient mechanism with little regulation [2]. During development, many pri-miRNAs are expressed but not efficiently processed to mature miRNA [3]. In healthy tissues, the ratio of pri- to mature miRNAs has been shown to be close to one, while in cancer cells, a large number of miRNA genes are transcribed but not processed to mature miRNA [4]. An expanding body of evidence supports a role for miRNAs in disease progression and the potential for epigenetic mechanisms to regulate miRNA expression [5,6]. Epigenetics is the heritable modification of gene expression without changes in the DNA sequence. The importance of regulated epigenetic information is highlighted by the disruption of multiple epigenetic marks in various disease states, including cancer, which is commonly associated with deregulation of DNA methylation, histone modifications and miRNA expression [7]. Cytosine methylation of CpG islands associated with gene promoters is a well-studied epigenetic mark, and aberrant DNA hypermethylation in combination with altered histone architecture is a common hallmark of neoplastic cells [8]. Histone acetylation at lysine 9 (H3K9Ac) is associated with an active chromatin state and changes in its global patterning are linked to poor prognosis in multiple cancers [9]. MiRNAs have been shown to function as tumor suppressors and oncogenes, through the targeting of essential elements of cellular growth, proliferation and apoptotic pathways [10]. Genomic loci encoding miRNAs can become epigenetically remodelled in cancer, however the extent and effect of these changes in cancer remains to be elucidated [6].

We argue that a minority of miRNA genes, critical to regulation of cellular growth, proliferation, or survival, gain epigenetic modifications to enable persistent enhancement or inhibition of their activity in order to maintain a neoplastic phenotype. Using an integrative approach combining primary transcription, genome-wide DNA methylation and H3K9Ac patterns with mature miRNA levels, we identified a number of miRNA genes that showed epigenetic remodeling in cancer in combination with de-regulation of both the primary and mature miRNA. This demonstrates that integration of multiple methods of analysis may provide a reliable method for the identification of epigenetically regulated miRNAs.

## Results and Discussion

**Figure 1:**
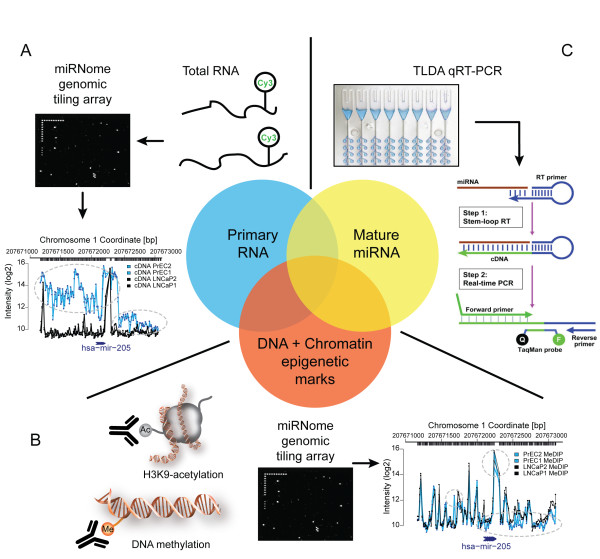
**Schematic displaying experimental discovery pipeline used for identification of miRNAs epigenetically deregulated in cancer**. To identify epigenetically regulated miRNAs, we employed an integrative approach assessing changes in epigenetic marks, primary miRNA transcript, and mature miRNA. (**A, B**) Custom tiling arrays probing all miRNA loci (miRBase12.0, 668 miRNA loci) were used to assay primary miRNA transcription and epigenetic marks (H3K9Ac and DNA methylation) from PrEC and LNCaP cells. Example tiling array intensity plots are shown of the 2000 bp region spanning the miR-205 gene for both expression (**A**), and DNA methylation (**B**), showing the chromosomal coordinate (x-axis) and the hybridization intensity (y-axis), with regions displaying significant differences highlighted. (**C**) Global mature miRNA expression levels in PrEC and LNCaP cells +/- 5-Aza-CdR treatment (3 μM LNCaP, 1 μM PrEC; 72 hours) as determined by TLDA qRT-PCR (368 miRNAs).

We proposed that epigenetically de-regulated miRNAs in cancer would show three characteristics: first, differential expression at the primary transcript level, second, differential mature miRNA expression, and finally an associated change in one or more epigenetic marks. To eliminate post-transcriptionally regulated miRNAs that showed accumulation of epigenetic marks independent of their transcription, an integrative pipeline that assessed all three criteria was developed (Figure 1).

Normal prostate epithelial (PrEC) and prostate cancer (LNCaP) cells were used as a model system to test this integrative pipeline in the discovery of epigenetically de-regulated miRNAs in cancer. Custom high-density NimbleGen tiling arrays covering miRNA loci from miRBase v12.0 were designed and used to probe total RNA to determine transcription levels, and DNA from methylated-DNA immunoprecipitation (MeDIP) and H3K9Ac immunoprecipitation to investigate epigenetic changes. The oligonucleotide probes of these arrays have an average length of 60nt, precluding interference of the mature 20-23nt miRNAs, thus assaying the full-length primary transcripts. Plotting the array probe intensities against chromosomal coordinates generated high-resolution maps of expression and epigenetic mark enrichment across each locus (plots for miR-205 are shown in Figure 1A &1B as examples). To generate lists of loci significantly changed in LNCaP cells for each assay, we selected those that were consistently changed in the cancer cell line within 500 bp of the center of the miRNA gene. For our comparisons, we limited the number of miRNAs to those that were assayed by both TaqMan^® ^Low Density Arrays (TLDA) (Figure 1C) and tiling array: 341 miRNAs in total. All changes are articulated as LNCaP with respect to PrEC.

**Figure 2:**
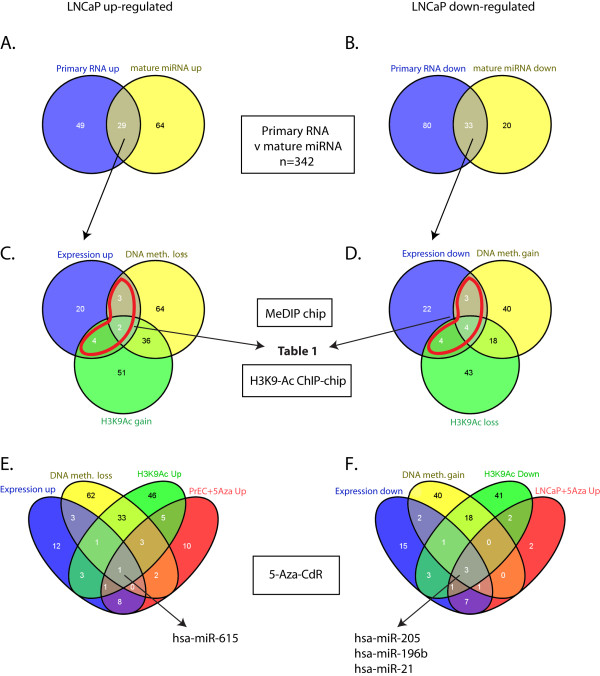
**Progressive steps in the identification of epigenetically regulated miRNAs in prostate cells. **Venn diagrams show the number of miRNA loci changed in LNCaP (L) with respect to PrEC (P) cells called significant by tiling arrays analysis using a t-statistic ≤-2 or ≥2 for regions +/-500 bp from the relevant miRNA. (**A, B**) Overlap of pri- and mature miRNA expression levels identified by tiling array and TLDA, respectively (TLDA miRNA expression data was normalized to RNU48, using a significance cutoff at p < 0.05 and ≥1.5 -fold change). Concomitant changes in active (H3K9Ac) or repressive (DNA methylation) epigenetic marks were then considered for miRNAs displaying significantly changed expression. (**C, D**) miRNA loci significantly changed in MeDIP-chip and H3K9Ac ChIP-chip tiling array analysis. (**E, F**) Four-way Venn diagrams showing the overlap of tiling array data with mature miRNA level upon 5-Aza-CdR treatment. MiR-615 lies at the four-way intersection indicating epigenetic up-regulation and miR-205, miR-196b and miR-21 are at the intersection of epigenetic down-regulation in LNCaP cells.

Looking at those loci whose primary transcript repression or activation in LNCaP was associated with a significant change in the mature miRNA, we identified 29 upregulated and 33 downregulated miRNAs (Figure 2A,B). We then looked for concomitant epigenetic changes at these loci in the tiling array data. A high proportion of the upregulated (31%; 9/29) and downregulated loci (33%; 11/33) showed an overlap with at least one of these epigenetic marks (Figure 2C,D). The majority (18/20) of these miRNAs have previously been identified as deregulated in various cancers, and five with prior evidence for epigenetic regulation (Table 1). A concordant change in all assays; expression, DNA methylation, and H3K9Ac restricted the total number of candidate miRNAs to 2/29 up- and 4/33 downregulated (Figure 2C,D). Given the large number of possible epigenetic modifications (for a recent review see [6]), it is possible that many of the differentially expressed loci that show no change in DNA methylation or H3K9Ac could undergo changes in other epigenetic marks. Furthermore, miRNAs may be regulated by DNA methylation at CpG islands outside the 2000 bp regions interrogated in this analysis.

**Table 1:** Candidate epigenetically regulated miRNAs

			Tiling array			Mature miRNA fold change						Reference
**Name**	**Chr**	**Str**	**RNA**	**DNA meth**.	**H3K9Ac ChIP**	**L v P**	**p**	**L v 5Aza**	**p**	**P v 5Aza**	**p**	**Epi./Cancer**

*MIRLET7c*	21	+	34.26	-0.14	3.07	54.948	*	0.986		1.505		-/[33]
*MIR141*	12	+	7.2	-0.46	4.31	2.928	*	2.235	*	1.110		[34]
*MIR15b*	3	+	11.41	-4.65	-4.02	2.071	*	0.616		0.986		-/[35]
*MIR16-2*	3	+	10.52	-3.6	-2.54	2.713	*	1.424	*	0.742		-/[36]
*MIR423*	17	+	8.91	-0.81	2.39	4.857	*	0.774		2.445	*	-/[37]
*MIR491*	9	+	9.03	-2.08	0.83	5.352	*	1.117		1.301		-/[38]
***MIR615***	12	+	4.03	-3.45	5.5	1.9E+03	*	1.231		32.672	*	-/-
*MIR618*	12	-	4.18	2.44	5.73	4.1E+02	*	1.945	*	1.385		-/[39]
*MIR99a*	21	+	26.52	-2.9	3.72	2.329	*	1.035		0.908		-/[40]

*MIR130a*	11	+	-7.19	-1.92	-3.85	0.004	*	2.532	*	1.102		-/[41]
*MIR135b*	1	-	-3.93	2.8	-7.82	0.003	*	0.337		1.828		-/[42]
*MIR137*	1	-	-3.81	1.19	-3.41	0.016	*	2.173		5.897		[43]
***MIR196b***	7	-	-11.26	2.07	-5.05	0.356	*	1.591	*	0.829		[21]/[44]
***MIR205***	1	+	-26.47	2.37	-12.09	1.2E-05	*	1.1E+02	*	1.659		[13]/[15]
***MIR21***	17	+	-21.68	13.78	-12.98	0.637	*	2.362	*	3.272	*	[19]/[18]
*MIR24-1*	9	+	-5.64	3.73	-0.27	0.057	*	1.569	*	1.231		-/[45]
*MIR31*	9	-	-2.79	-9.75	-3.04	1.7E-06	*	1.357		0.959		-/[46]
*MIR376a-2*	14	+	-5.98	2.96	2.59	0.020	*	4.857		2.888		-/[47]
*MIR424*	X	-	-3.94	1.95	-2.56	0.259	*	0.883		4.287	*	-/[39]
*MIR654*	14	+	-5.42	2.57	4.14	0.136	*	1.357		0.295	*	-/-

Table listing significantly changed miRNA genes identified in this study. Values represent relative change in LNCaP compared to PrEC. Tiling array t-statistics represent the significance of the average difference for each miRNA, with increasing values indicating higher significance level. Mature miRNA levels are given as -fold change with the associated p-value. The four miRNAs found to be significant in all our criteria are highlighted in bold type. Chr: Chromosome, Str: Strand, meth: DNA-methylation, L: LNCaP, P: PrEC, 5Aza: treatment with 5-Aza-CdR, p *: p-value < 0.05, Epi./Cancer: publication indicating epigenetic/cancer regulation.

In order to perform an independent test for miRNAs regulated by DNA methylation, we treated LNCaP and PrEC cells with the drug 5-Aza-CdR, an inhibitor of DNA methylation, and analysed their expression by TLDA. Restricting our list to miRNAs that were significantly deregulated between PrEC and LNCaP we found 30 upregulated by 5-Aza-CdR in PrEC and 16 in LNCaP. Of the miRNA loci showing epigenetic activation by the three criteria of primary transcript expression, DNA methylation loss and H3K9Ac gain, only miR-615 showed 5-Aza-CdR induced reactivation in the normal prostate PrEC cells (Figure 2E). MiR-205, miR-196b and miR-21 satisfied all three criteria for epigenetic repression, and LNCaP 5-Aza-CdR reactivation (Figure 2F).

MiRNA genes showing non-canonical epigenetic regulation were also observed. That is, miRNAs showing up regulation of primary and mature miRNA expression, with a concomitant gain of DNA methylation or loss of H3K9Ac, or vice versa. Twelve miRNAs showed activation and gain of repressive epigenetic marks and six (including the chromosome 14 miRNA cluster) showed repression in combination with active chromatin (Additional file 1, Table S1). None of these miRNAs showed significant up regulation upon treatment with 5-Aza-CdR. Several miRNAs were repressed in the presence of the demethylating drug, suggesting the repression of these loci may be dependent on genes that are themselves subject to regulation by DNA methylation.

**Figure 3:**
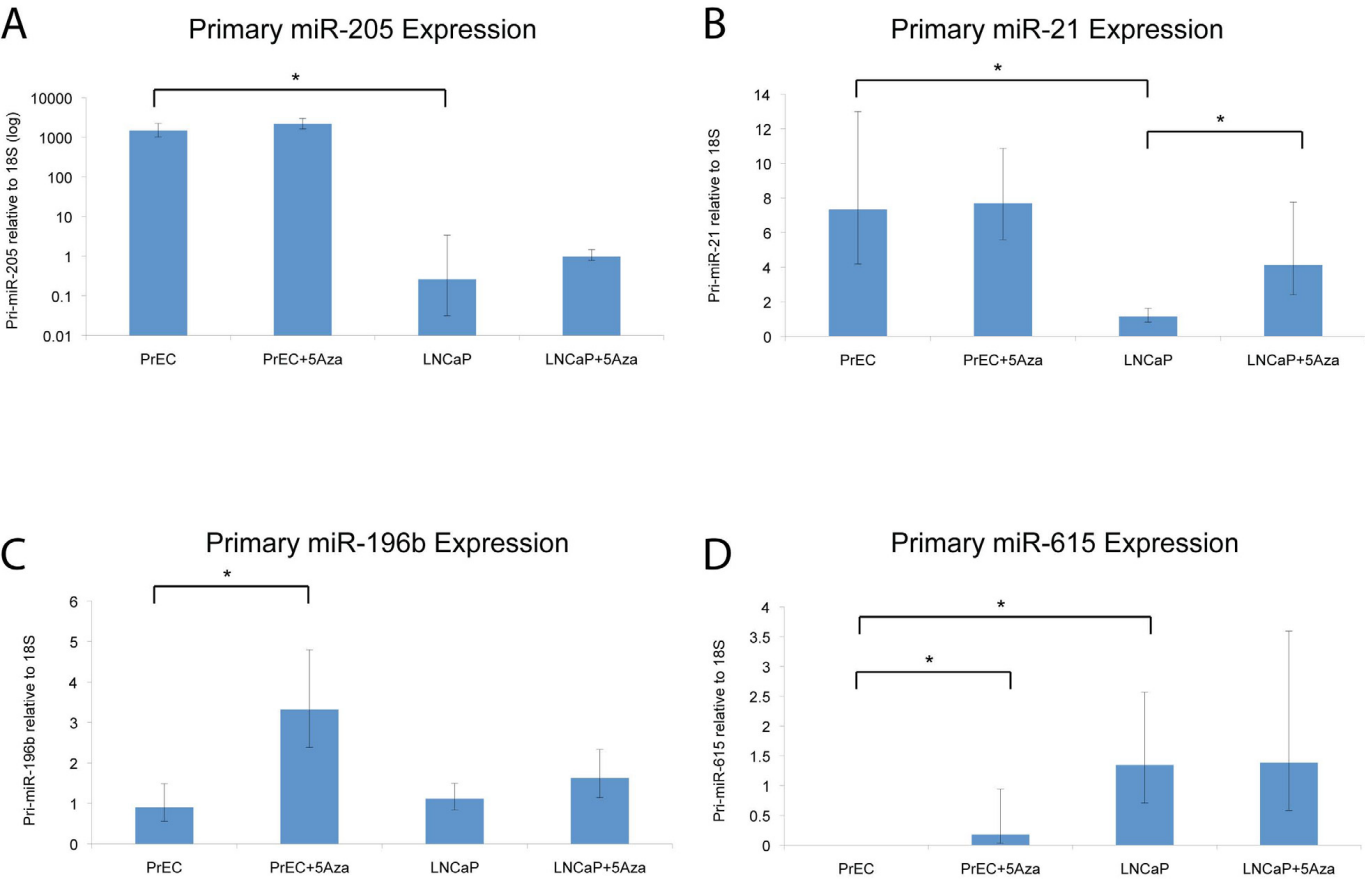
**Vailidation of pri-miRNA expression**. Quantitative TaqMan^® ^RT-PCR of pri-miRNA expression in LNCaP and PrEC cells +/- 5-Aza-CdR treatment (n = 6, +/-SD, *p <0.05). Pri-miRNA RT-PCR assays followed established TaqMan^® ^workflows (Applied Biosystems). Briefly, total RNA was used for first-strand cDNA synthesis using 1 μg RNA Superscript reverse transcriptase (Invitrogen). cDNA was then subjected to 40 cycles of amplification using an ABI7900 instrument (Applied Biosystems). Data was normalized to 18S levels (2^-dCt^).

To validate the changes in primary miRNA expression, we performed pri-miRNA TaqMan^® ^qPCR on the four miRNAs that met all our criteria. These assays confirmed the repression of pri-miR-205 and pri-miR-21, and over expression of pri-miR-615 in LNCaP cells (Figure 3A,B,D). There was no significant change in pri-miR-196b between LNCaP and PrEC, however treatment with 5-Aza-CdR led to overexpression in PrEC cells (Figure 3C). 5-Aza-CdR led to overexpression of pri-miR-21 and pri-miR-615 in LNCaP, as would be predicted by their DNA methylation status (Figure 3B,D). However, reactivation of pri-miR-205 by 5-Aza-CdR was not significant (Figure 3A).

To verify changes in DNA methylation we analysed recently published methyl-CpG binding domain-based capture (MBDCap) data from LNCaP and PrEC cells [11]. MBDCap preferentially captures DNA methylated double-stranded DNA which is then eluted for high-throughput sequencing [12]. We examined the number of sequencing reads that mapped to within 300 bp of the selected miRNA loci. In agreement with the tiling array data, there was enrichment of sequencing reads at the *MIR196b *locus (Additional file 1, Figure S1). *MIR205 *and *MIR21 *had increased reads in LNCaP over PrEC, however there was also an increase in the input DNA controls. *MIR615 *showed a low number of sequencing events in both LNCaP and PrEC and input controls (Additional file 1, Figure S1).

The identification of miR-205 as an epigenetically repressed miRNA is consistent with a previous study that examined chromatin modifications in prostate cells using genome-wide promoter arrays, which revealed a gain of the repressive H3K27-trimethylation mark and loss of the active H3K4-trimethylation mark at the *MIR205 *locus in prostate cancer cells [13]. Furthermore, we recently reported that *MIR205 *locus is silenced and gains promoter hypermethylation and repressive chromatin marks in invasive bladder tumors and undifferentiated bladder cell lines [14]. Two expression array studies have also identified miR-205 as repressed in prostate cancer [15,16]. While the repression of pri-miR-205 was validated by qPCR, the lack of significant change in DNA methylation observed in the MBDCap high-throughput sequencing data could be due to differences between the techniques. Both *MIR205 *and *MIR21 *reside in regions of low CpG density, whereas *MIR196b *and *MIR615 *are located in regions of high CpG density, which may bias the purification efficiencies. Variations between pri-miRNA and array results, such as with pri-miR196b, could be accounted for by differences in assay design. An advantage of the tiling array approach is the high-density coverage of all loci. PCR typically provides improved dynamic range over array analysis, however, the efficiency of a reaction can be limited by location of the primers - as may be the case with pri-miRNA transcripts that have yet to be exhaustively mapped. MiR-21 shows significant epigenetic repression in our array and qPCR analyses, contradictory to its well-established role as an oncogenic miRNA that is overexpressed in cancer [17], however recent evidence has also shown that miR-21 knockdown elicited no change in proliferative or invasive properties in prostate cancer cells [18].

The potential for epigenetic regulation of *MIR21 *has previously been shown in ovarian cancer, where treatment with 5-Aza-CdR leads to miR-21 overexpression [19], consistent with hypermethylation of the locus. It is possible that epigenetic mechanisms may play a role in context dependent regulation of miR-21 expression, and it would be interesting to examine the prevalence of epigenetic marks at in a cohort of clinical prostate cancer samples. The epigenetic regulation of both *MIR615 *and *MIR196b *is particularly intriguing as both genes are encoded from CpG islands located within *HOX *gene clusters. *MIR196b *has recently been shown to be hypomethylated in acute lymphoblastic leukemia [20] and gastric cancer [21] but to the best of our knowledge, the epigenetic regulation of *MIR615 *is a novel discovery. *MIR615 *is located within the *HOXC *cluster on chromosome 13, which we previously have shown to be epigenetically deregulated in LNCaP cells in long range epigenetically silenced domains [22]. There is evidence that *HOX *associated miRNAs are essential for development and can function by targeting *HOX *messenger RNAs [23]. Furthermore, *HOX *genes have been reported to be involved in abnormal development and malignancy in numerous cancers via both genetic and epigenetic mechanisms [24].

There are inherent limitations to the various available global epigenetic discovery techniques [11]. Immunoprecipitation based techniques can be affected by antibody sensitivity and MeDIP can be biased towards regions of high CpG content, leading to increased false-negatives at GC-poor sites [25]. In addition, treatment with demethylating drugs such as 5-Aza-CdR can uncover false positives through activation or repression of downstream targets [26]. A multi-platform approach can help rule out many of these false positive results, but the problem of false negatives remains. Here we show that using miRNA primary transcript data enables leverage of the known mechanism of post-transcriptional regulation of miRNAs as an additional means of epigenetic marker discovery. Epigenetic marks such as DNA methylation possess several advantages as biomarkers; DNA is more robust than RNA, can be isolated from formalin fixed paraffin-embedded archival samples and DNA methylation provides a positive readout for genes or miRNAs that are downregulated [27].

## Conclusions

We found that up to 33% of transcriptionally deregulated miRNA loci displayed concordant DNA methylation and H3K9 acetylation patterns. Extending the analysis to additional epigenetic marks, such as the methylation of H3K4, H3K9 and H3K27 and acetylation at H3K14 will increase the number of identified loci. The use of tiling arrays in this study enabled analysis within 2000 bp of a miRNA gene and the increasing availability of high-throughput sequencing technology will allow this approach to be extended genome wide. The prevalence of miRNAs that show contradicting expression and epigenetic marks suggests that there is a subset of genes that are subject to a complex pattern of regulation, where specific marks may recruit activators or repressors to a promoter. We propose that in cancer, a change in primary transcript expression at miRNA loci provides an important first step in the identification of those miRNAs that display variant epigenetic marks. Overlapping these loci with change in mature miRNA restricts analysis to miRNAs that are functionally relevant and that provide possible selective advantage to the cancer cell. Further discovery of DNA hyper- and hypo-methylated miRNAs in cancer using this type of integrative discovery pipeline should assist in identifying loci that may provide reliable novel diagnostic tools.

## Methods

### Cell Lines and Culture

LNCaP cells were propagated in T-media as described previously [28]. PrEC cells were propagated in PrEGM (Lonza, Switzerland). 5-Aza-2'-deoxycytidine (5-Aza-CdR) (Fluka, MO, USA) treatment was performed for 72 hours with fresh media and drug added at 24 and 48 hours. We optimized 5-Aza-CdR treatment of LNCaP and PrEC cells at 3 μM and 1 μM, respectively, based on reactivation of the imprinted gene *IGF2 *and the prostate cancer hypermethylated gene *GSTP1 *in response to treatment, as described previously [28]. Total RNA was isolated using Trizol reagent according to supplier's protocol (Invitrogen, CA, USA). Genomic DNA was extracted from untreated LNCaP and PrEC using the Mammalian Blood and Tissue Kit (Qiagen, Germany).

### TaqMan^® ^Low-Density Arrays (TLDA)

TLDA v1.0 Early Access (Applied Biosystems, CA, USA) assays for 368 miRNAs were performed as per manufacturer's protocols. In brief, 1 μg total RNA from 5-Aza-CdR treated and untreated LNCaP and PrEC biological duplicates was reverse transcribed with pools of miRNA specific RT-primers, and the resultant cDNA was subjected to real-time reverse transcription quantitative (qRT)-PCR using specific forward primers and TaqMan^® ^fluorescent probes in an array format. RNU48 was selected as the most reliable control for expression normalization. Initial analyses were performed using SDS software (Applied Biosystems). Statistical tests for changes in expression (via Cts) were performed using moderated t-tests without multiple testing corrections using the R limma package [29]. Mature miRNAs were deemed differentially expressed in LNCaP compared to PrEC at p <0.05 and fold change of ≥1.5.

### Expression Tiling Arrays

Total RNA (6 μg) from untreated LNCaP and PrEC duplicates was reverse transcribed with random primers and aminoallyl- conjugated dUTP. cDNA products were precipitated and resuspended with mono-reactive Cy3/5 dye (GE Healthcare, UK), and the products were purified with Fairplay columns (Stratagene, TX, USA). Labeling efficiency was determined spectrophotometrically. Hybridizations for untreated LNCaP and PrEC were repeated in two formats to test a method to minimize probe GC bias: competitive hybridization with fluorescently labeled gDNA in the Cy5 channel, and in a dye swap arrangement. The biological repeats were highly reproducible (Pearson correlations: LNCaP 0.94 and PrEC 0.96) and co-hybridization showed no correction to probe binding efficiency (data not shown).

### Tiling Array MeDIP-chip/H3K9Ac-ChIP-chip

#### MeDIP

4 μg of genomic DNA from untreated LNCaP and PrEC biological duplicates was sonicated, then denatured followed by incubation with Anti-5-Methylcytosine antibody (Calibiochem) overnight at 4°C. The antibody/DNA complexes were affinity purified using protein A/G agarose beads (Santa Cruz Biotechnology). Products were washed, eluted and ethanol precipitated. Efficiency of immunoprecipitation was confirmed by PCR at three loci; centromeric chromosome 16, *GSTP1 *and *GAPDH*, and compared with no-antibody and input controls, as described previously [30]. Biological duplicates were highly reproducible (Pearson correlation: LNCaP and PrEC 0.96).

#### H3K9Ac ChIP

LNCaP and PrEC cells were formaldehyde fixed in duplicate prior to SDS lysis and sonication. Immunoprecipitation (IP) was performed overnight at 4°C with an anti-acetyl H3K9 antibody (Millipore, CA, USA). DNA was eluted with 0.1M NaHCO3, 1% SDS elution buffer and reverse cross-linked. IPs and input controls were purified by phenol chloroform extraction and ethanol precipitation, as described previously [31], duplicate IP reactions were then pooled to ensure sufficient material for array analysis. IP DNA from MeDIP and H3K9Ac were labeled as per the manufacturers protocol (NimbleGen, WI, USA). Briefly, 1 μg IP or input DNA was labeled with Cy-dye random nonamers (Trilink Biotechnologies, CA, USA) using Klenow fragment (New England Biolabs, MA, USA), precipitated, and resuspended in hybridization buffer.

### Tiling Arrays

We designed probes for 384 K feature tiling arrays (NimbleGen) to cover the positive strand for the following genomic regions: all human miRNAs listed in mirBASE v12.0 +/-2000 bp; HOX A,B,C and D gene clusters; the chromosome 14 Dlk1 - Dio3 - miRNA cluster region; and hybridization control regions. Array hybridization was performed for 16 hours with 6 μg labeled cDNA with hybribization buffer and alignment oligonucleotides (NimbleGen). Arrays were washed as per the manufacturers protocol and scanned using a GenePix 4000B scanner (Molecular Devices, CA, USA). To screen for miRNA loci that were significantly changed between LNCaP and PrEC cells, we fitted a linear model to the quantile-normalized probe-level data for all probes within 500 bp of the center of a miRNA gene. From the linear model fit, the effect of interest is the average change between PrEC and LNCaP across all probes, after adjusting for probe-specific effects. The t-statistics shown in Table 1 represent the significance of this average difference for each miRNA. MiRNAs were deemed statistically significant using a t-statistic cutoff of ≤-2 or ≥2. Venn diagrams were drawn using Venny [32], and restricted to overlaps for those miRNAs analysed by TLDA. Results of all array experiments are listed in Additional file 2.

### Pri-miRNA Real-Time PCR

We used Applied Biosystems pri-miRNA stem-loop specific TaqMan assays to quantify pri-miRNAs, as per the manufacturers protocol. Briefly, first-strand cDNA was synthesized using 10 ng DNase (RNase free DNase, Qiagen) treated total RNA, or no-template controls, and Superscript reverse transcriptase (Invitrogen). Real-time PCR reactions for each miRNA (10 μl volume) included 4 μl of RT product, were performed in triplicate and included no-template and PCR controls using an ABI7900 PCR system (Applied Biosystems). Reactions performed under the following conditions: 95°C for 10 minutes, followed by 40 cycles at 95°C for 15 seconds and 60°C for 1 minute. Relative quantitative levels of individual miRNAs were determined using 18S for normalization, and statistically significant differences were analysed using the students t-test.

## Abbreviations

miRNA: microRNA; TLDA: TaqMan Low Density Array; ChIP: Chromatin Immunoprecipitation; bp: base pairs; 5-Aza-CdR: 5-Aza-2'-deoxycytidine; IP: immunoprecipitation.

## Authors' contributions

TH and SJC conceived the project. TH wrote the manuscript, cultured cells, performed array experiments and analysis. TS cultured cells and performed ChIP, MeDIP and TLDA experiments. EDW performed tiling array experiments. SB performed MeDIP experiments. ALS, DS and MDR performed data analyses. All authors read and approved the final manuscript.

## Supplementary Material

Additional file 1 **Table S1: MiRNAs showing non-canonical epigenetic regulation**. Table listing miRNA loci and genes showing significant non-canonical epigenetic regulation. Values are expressed as changes in LNCaP relative to PrEC. Tiling array t-statistics represent the significance of the average difference for each miRNA, with increasing value indicating more significant change. Mature miRNA levels are given as fold change with the associated p-value. Chr: Chromosome, Str: Strand, meth: DNA-methylation, L: LNCaP, P: PrEC, 5Aza: treatment with 5-Aza-CdR, p *: p-value <0.05, Epi./Cancer: publication indicating epigenetic/cancer regulation. Chr14 cluster data includes: *MIR127, MIR323, MIR376a-2, MIR432, MIR376a-1, MIR654, MIR382, MIR487b, MIR134, MIR487b*, and *MIR411*. Click here for file

Additional file 2 **Figure S1: Validation of DNA methylation in selected miRNA loci**. Analysis of published DNA methylation enrichment data at selected miRNA loci from *Robinson et al. *[11]. Graph displays absolute number of MBDCap high throughput sequencing reads within 300 bp of the miRNA locus. Click here for file
