# Supplementary material for: Discovery pipeline for epigenetically deregulated miRNAs in cancer: integration of primary miRNA transcription
Source: BMC Genomics. 2011 Jan 21;12:54. doi: 10.1186/1471-2164-12-54 (PMC3037319; doi:10.1186/1471-2164-12-54)

## Additional File 1, Table S1 - miRNAs showing inverse expression and epigenetic regulation.

|  |  |  | **Tiling Array** | | | **Mature miRNA fold change** | | | | | |
| --- | --- | --- | --- | --- | --- | --- | --- | --- | --- | --- | --- |
| **Name** | **Chr** | **Str** | **RNA** | **DNA meth.** | **H3K9Ac ChIP** | **L v P** | **p** | **L v 5Aza** | **p** | **P v 5Aza** | **p** |
| *MIR15b* | 3 | + | 11.41 | -4.65 | -4.02 | 2.071 | * | 1.625 |  | 1.014 |  |
| *MIR16-2* | 3 | + | 10.52 | -3.60 | -2.54 | 2.713 | * | 0.702 | * | 1.347 |  |
| *MIRLET7d* | 9 | + | 9.97 | 1.45 | -3.30 | 5.389 | * | 1.072 |  | 0.570 | * |
| *MIR101-2* | 9 | + | 7.56 | 0.95 | -3.48 | 7.413 | * | 0.624 |  | 0.426 |  |
| *MIR193b* | 16 | + | 5.28 | 2.89 | -0.86 | 13.177 | * | 1.064 |  | 0.130 | * |
| *MIR378* | 5 | + | 4.29 | 5.48 | -5.41 | 2.250 | * | 1.087 |  | 1.231 |  |
| *MIR618* | 12 | - | 4.18 | 2.44 | 5.73 | 407.315 | * | 0.514 | * | 0.722 |  |
| *MIR148b* | 12 | + | 3.93 | 0.41 | -5.07 | 4.563 | * | 0.946 |  | 0.566 | * |
| *MIR365-1* | 16 | + | 3.69 | 2.15 | -0.40 | 3.784 | * | 0.953 |  | 0.423 | * |
| *MIR500* | X | + | 2.86 | 2.78 | 1.08 | 64.893 | * | 1.424 |  | 0.933 |  |
| *MIR342* | 14 | + | 2.59 | 4.21 | -1.86 | 4.438 | * | 0.865 |  | 0.660 | * |
| *MIR30d* | 8 | - | 2.28 | -1.44 | -3.01 | 2.990 | * | 0.655 |  | 0.620 |  |
| *MIR31* | 9 | - | -2.79 | -9.75 | -3.04 | 0.000 | * | 0.737 |  | 1.042 |  |
| *MIR23a* | 19 | - | -2.88 | -2.14 | 0.95 | 0.054 | * | 0.493 |  | 0.674 |  |
| *MIR187* | 18 | - | -3.42 | -4.78 | 1.99 | 0.013 | * | 0.002 | * | 0.123 |  |
| *MIR330* | 19 | - | -4.28 | -2.58 | -0.16 | 0.274 | * | 0.379 |  | 0.785 |  |
| *chr14 cluster* | 14 | + | -4.35 | -3.34 | 4.26 | 0.054 | * | 0.468 |  | 0.898 |  |
| *MIR149* | 2 | + | -6.77 | -8.67 | 2.87 | 0.274 | * | 0.914 |  | 0.642 |  |

## Additional File 1, Figure S1 - Validation of DNA methylation in selected miRNA loci.


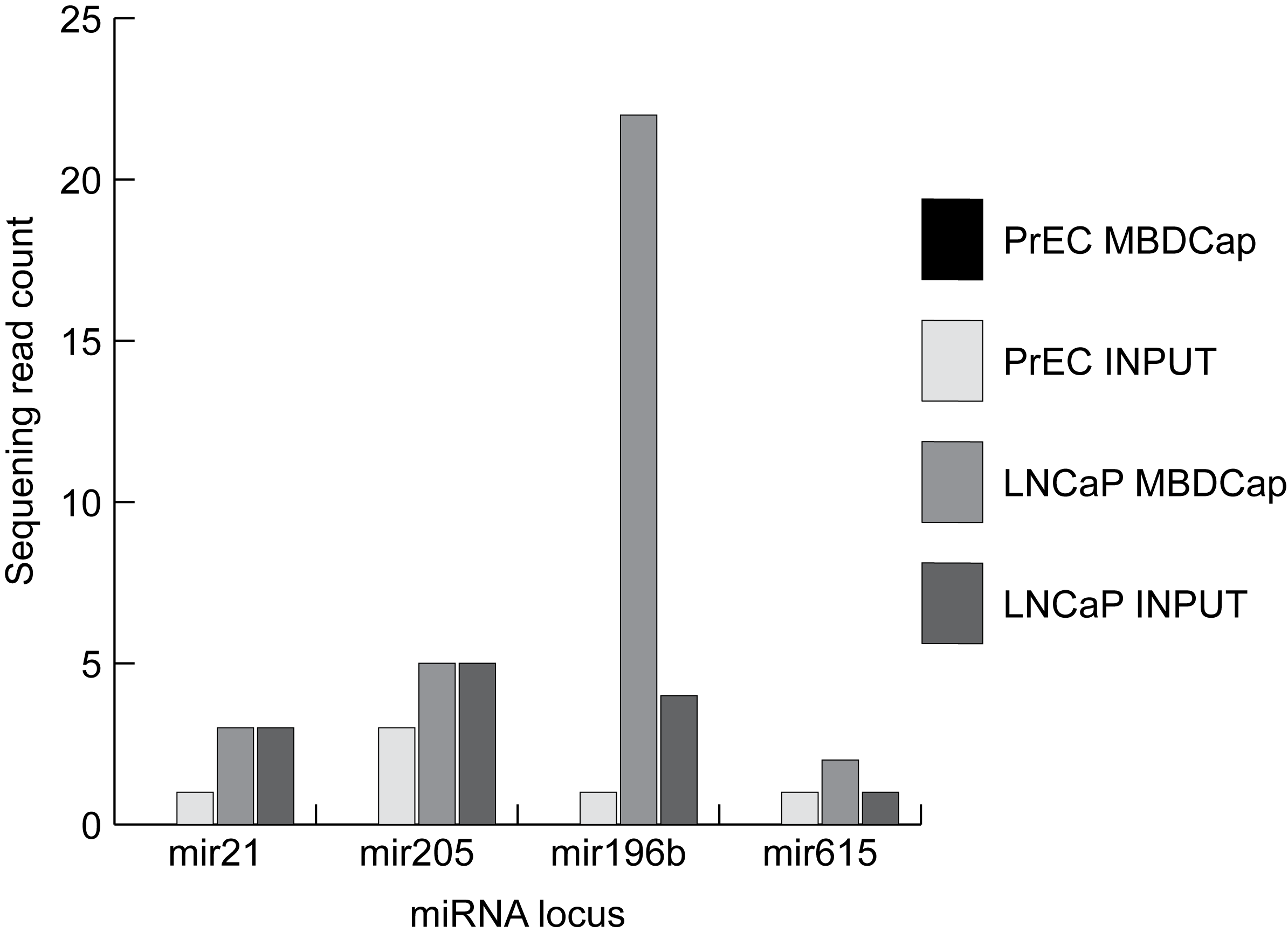

Supplement: Additional file 1 — Table S1: MiRNAs showing non-canonical epigenetic regulation. Table listing miRNA loci and genes showing significant non-canonical epigenetic regulation. Values are expressed as changes in LNCaP relative to PrEC. Tiling array t-statistics represent the significance of the average difference for each miRNA, with increasing value indicating more significant change. Mature miRNA levels are given as fold change with the associated p-value. Chr: Chromosome, Str: Strand, meth: DNA-methylation, L: LNCaP, P: PrEC, 5Aza: treatment with 5-Aza-CdR, p *: p-value <0.05, Epi./Cancer: publication indicating epigenetic/cancer regulation. Chr14 cluster data includes: MIR127, MIR323, MIR376a-2, MIR432, MIR376a-1, MIR654, MIR382, MIR487b, MIR134, MIR487b, and MIR411. [file 1471-2164-12-54-S1.DOC]
